# Immunization with Genetically Modified Trypanosomes Provides Protection against Transmissible Spongiform Encephalopathies

**DOI:** 10.3390/ijms231810629

**Published:** 2022-09-13

**Authors:** Gianna Triller, Dimitrios A. Garyfallos, F. Nina Papavasiliou, Theodoros Sklaviadis, Pete Stavropoulos, Konstantinos Xanthopoulos

**Affiliations:** 1 Laboratory of Lymphocyte Biology, The Rockefeller University, New York, NY 10065, USA; 2 Cambridge Institute of Therapeutic Immunology & Infectious Disease (CITIID), University of Cambridge, Puddicombe Way, Cambridge CB2 0AW, UK; 3 Division of Immune Diversity, Deutsches Krebsforschungszentrum, 69120 Heidelberg, Germany; 4 Laboratory of Pharmacology, School of Pharmacy, Aristotle University of Thessaloniki, 54124 Thessaloniki, Greece; 5 Institute of Applied Biosciences, Centre for Research and Technology Hellas, 57001 Thermi, Greece

**Keywords:** prion diseases, trypanosomes immunization, active immunization, neuroprophylaxis

## Abstract

Transmissible spongiform encephalopathies are incurable neurodegenerative diseases, associated with the conversion of the physiological prion protein to its disease-associated counterpart. Even though immunization against transmissible spongiform encephalopathies has shown great potential, immune tolerance effects impede the use of active immunization protocols for successful prophylaxis. In this study, we evaluate the use of trypanosomes as biological platforms for the presentation of a prion antigenic peptide to the host immune system. Using the engineered trypanosomes in an immunization protocol without the use of adjuvants led to the development of a humoral immune response against the prion protein in wild type mice, without the appearance of adverse reactions. The immune reaction elicited with this protocol displayed in vitro therapeutic potential and was further evaluated in a bioassay where immunized mice were partially protected in a representative murine model of prion diseases. Further studies are underway to better characterize the immune reaction and optimize the immunization protocol.

## 1. Introduction

Transmissible spongiform encephalopathies (TSEs) are neurodegenerative diseases sharing a common pathogen, termed a prion, which consists mainly or exclusively of the disease-associated isoform (PrP^Sc^) of the cellular prion protein (PrP^C^). These isoforms share the same primary structure, but differ in their conformation, with PrP^Sc^ being β-sheet enriched. This structural difference, most probably, gives rise to the observed differences in physicochemical and biological properties, including solubility and proteinase K resistance that the two isoforms display [1]. It is believed that upon infection, PrP^Sc^ dictates the conversion of host PrP^C^ molecules to PrP^Sc^, significantly amplifying the pool of disease associated molecules. These novel PrP^Sc^ molecules can in turn further propagate conversion events, leading to the appearance of the disease phenotype. Although the conversion mechanism has not yet been elucidated, and currently only computational models are available [2], it is believed that it plays a crucial role in disease pathogenesis.

The physiological role of the prion protein has not yet been elucidated, although many different functions have been attributed to the protein [3,4,5]. Of note, PrP KO mice do not develop any overt phenotype and appear normal, with the only undisputed phenotype being their resistance to infections with the TSE agent [6].

Human prion diseases include, among others, the various forms (sporadic, variant, familial, iatrogenic) of Creutzfeldt Jakob disease, Kuru and fatal familial insomnia. In animals, the most common forms of TSEs include scrapie in sheep, bovine spongiform encephalopathy (BSE) in cattle and chronic wasting disease (CWD) in cervids [7]. Despite fervent research, TSEs remain incurable, and there is a clear and unmet need to develop novel protective and/or therapeutic agents [8,9].

Many different immunoprophylactic and immunotherapeutic approaches against TSEs have been tested throughout the years, some of which proved to be very promising [10,11]. Passive immunization in a murine model of prion diseases significantly reduced prion infectivity and provided full protection against disease development [12]. Active oral immunization against prion diseases using a live attenuated *Salmonella typhimurium* strain, genetically modified to express either one or two copies of PrP, led to the development of mucosal immunity and protection against oral challenge in a murine prion model [13,14]. A similar approach was implemented in deer to protect against CWD, providing substantial elongation of the survival interval [15].

However, non-oral active immunization approaches provide only modest results. We and others have tried immunization with different forms of recombinant PrP, achieving only partial protection against disease progression [16,17,18,19]. In addition to using different forms of recombinant PrP, varied strategies have been implemented to further enhance immune responses. For example, animals were immunized with PrP peptides supplemented with CpG oligodeoxynucleotide [20], a combination of DNA and peptide vaccines [21], dendritic cells loaded with prion peptides [22,23], or viral-like particles expressing prion peptides [24,25]. The poor protection offered by the active immunization protocols has been attributed to the close resemblance between the normal and the pathogenetic isoform of the protein. This resemblance apparently leads to immune tolerance against PrP^Sc^, which hampers active immunization schemes against TSEs, and enables PrP^Sc^ to evade the immune system [26]. As a result, TSEs do not trigger immune responses and remain incurable, although passive immunization against PrP has shown great promise [27].

To trigger a potent immune response against PrP protein, we explored the possibility of using a genetically modified, inactivated protozoan parasite (*Trypanosoma brucei*) as an antigen carrier. When found in the bloodstream of mammalian hosts, *T. brucei* is covered by a dense coat composed of ~11 million identical, variable surface glycoprotein (VSG) molecules. VSGs are glycophosphatidylinositol-anchored and encoded by a large (>2000) repertoire of genes. However, only one VSG gene is active at any given moment. The VSG coat is strongly immunogenic and provides protection against the host immune system, by covering and rendering virtually inaccessible other invariant proteins “hidden” beneath it. At the same time, the coat is used as a decoy: it first elicits a robust antibody response, and then it is swapped for a new coat (a process termed antigenic variation), thus efficiently evading the just-established response [28,29].

We have previously shown that integration of an antigen within the highly repetitive form of the trypanosome’s VSG coat facilitates the antigen’s presentation to the host immune system, and leads to the initiation of a robust, humoral-only response by the host [30]. Based on this study, we investigated whether immunization of wild type mice with Trypanosomes expressing a PrP-specific peptide could elicit an antibody-based immune response offering protection against TSEs. Our data indicate that immunization with the engineered trypanosomes can circumvent immune tolerance against PrP, eliciting a strong immune response, which provides partial protection in an animal model of prion diseases.

## 2. Results

### 2.1. Generation of Engineered T. brucei with Prion Peptide Expressing Surface VSG

**Figure 1:**
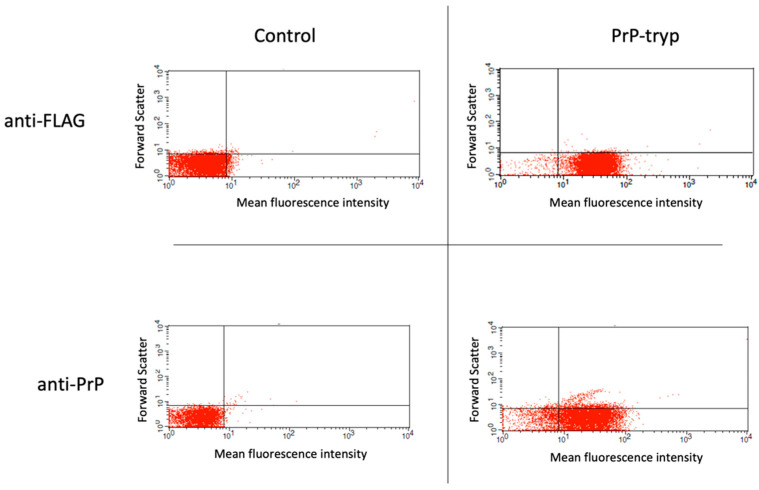
Flow cytometry to identify trypanosomes expressing the engineered variable surface glycoprotein (VSG). Following selection, stably transfected (PrP-tryp) and control (untransfected) *T. brucei* clones were stained with an anti-FLAG and an anti-PrP (6H4) antibody. A *T. brucei* clone with robust expression of the surface tag and the PrP peptide was identified.

Following the generation of different expression vectors encoding for the expression of the prion HFGNDWEDRYYRENMY peptide and an HA- or FLAG-tag on the VSG coat, *T. brucei* were transfected and assessed for their ability to grow in the presence of the selection agent. Transfection with approximately half the expression vectors prepared and tested (16/34) was stable, allowing for long-term culture in the presence of phleomycin. These stably transfected trypanosomes were further probed by flow cytometry for the surface expression of the FLAG or HA tag, and then for the prion peptide. A representative staining of a control (untransfected) and a stably transfected clone expressing the prion peptide is given in Figure 1. Of the 16 stably transfected trypanosome clones with the different expression vectors, efficient surface expression of the prion peptide was only achieved when the peptide was expressed in VSG2, in combination with the FLAG tag, when the distance between the peptide and the tag was maximal.

### 2.2. Immunization with the Trypanosomes Leads to the Production of Anti-PrP Antibodies

**Figure 2:**
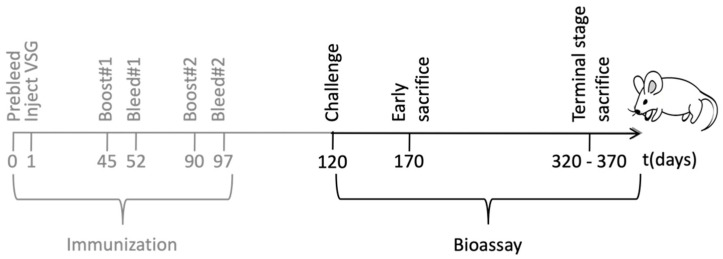
Schematic representation of the immunization and the bioassay. Female BALB/c mice were immunized with *T. brucei* expressing PrP-VSG (N = 11) or WT-VSG (N = 15). Serum was harvested at regular time points throughout the immunization and the possible protective role of the immunization was evaluated in a bioassay using a murine scrapie strain.

In order to characterize the immune response elicited by the engineered trypanosomes, groups of BALB/c mice were immunized with 10^7^ formalin-fixed trypanosomes, bearing either a PrP-VSG coat (PrP-tryp group, N = 11) or a wild type (WT)-VSG coat (Control group, N = 15). The trypanosomes were suspended in phosphate buffer saline (PBS) and administered intraperitoneally a total of three times in 45-day intervals. Serum was collected before administration (pre-bleed), and 7 days after the second and third administration (summarized in Figure 2 in grey).

**Figure 3:**
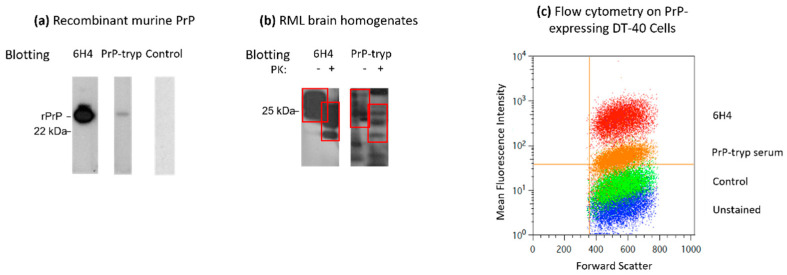
Characterization of the immune sera. Immune serum was used as the primary antibody in Western blots to detect recombinant murine PrP (**a**) or PrP in brain homogenates from terminally ill Rocky Mountains Laboratory (RML) mice (**b**). The immune serum recognizes recombinant PrP, as well as total PrP (PrP^C^ and PrP^Sc^) and PrP^Sc^ in brain homogenates. (**c**) The immune serum also recognized PrP expressed on the surface of DT-40 cells stably transfected to express PrP. PrP-tryp: serum from a mouse immunized with PrP-VSG expressing trypanosomes; Control: serum from a mouse immunized with WT-VSG expressing trypanosomes; 6H4: monoclonal anti-PrP antibody. Red boxes indicate the di-, mono- and unglycosylated forms of PrP. Data from a representative PrP-tryp and a control serum (second bleed) are depicted in all three panels.

Mice did not display any overt symptoms throughout the study, indicating that immunization with formalin-fixed trypanosomes is safe. Sera collected from the immunized mice was used as the primary antibody in Western blots and flow cytometry against recombinant murine PrP. Sera from the first bleed, collected 7 days after the second trypanosomes administration, showed only minimal reactivity by Western blot and flow cytometry. However, after the second boost, sera from the PrP-tryp immunized mice, but not the control mice, successfully recognized the recombinant form of the protein (Figure 3a). These sera were also used to blot PrP^Sc^ in brain homogenates from terminally ill Rocky Mountains Laboratory (RML) mice (Figure 3b). The sera recognized the un-, mono- and di-glycosylated forms of the prion protein both in proteinase K-untreated, which contained PrP^C^ and PrP^Sc^, and proteinase K-treated samples, which are enriched in PrP^Sc^, confirming that the immune sera can interact not only with the recombinant, but also the denatured native form of the murine prion protein.

To further validate the therapeutic and protective potential of the immune sera generated, we evaluated whether the sera were able to recognize PrP in its native form by flow cytometry, as expressed on the surface of eukaryotic cells. To this end, DT-40 cells stably expressing PrP on the cell surface were stained with the immune sera using a secondary fluorescence-tagged anti-mouse IgG antibody, to determine binding. Similar to the monoclonal anti-PrP antibody (6H4), sera from the PrP-tryp immunized mice successfully recognized the protein (Figure 3c).

### 2.3. Immunization Provides Partial Protection in a Murine Model of Prion Diseases

To evaluate the possible protective role of the elicited immune response against prion diseases, the PrP-tryp (N = 11) and the control mice (N = 15) were challenged intraperitoneally (i.p.) with the RML murine scrapie strain, 30 days after completion of the immunization protocol (Figure 2). Mice were euthanized at two timepoints: 50 days after the RML challenge, to determine differences in early PrP^Sc^ accumulation in the periphery (four mice per group), or at each mouse’s terminal stage (defined by severe weight loss with dyskinesia or paralysis), to evaluate any protective effect provided by the immunization protocol.

**Figure 4:**
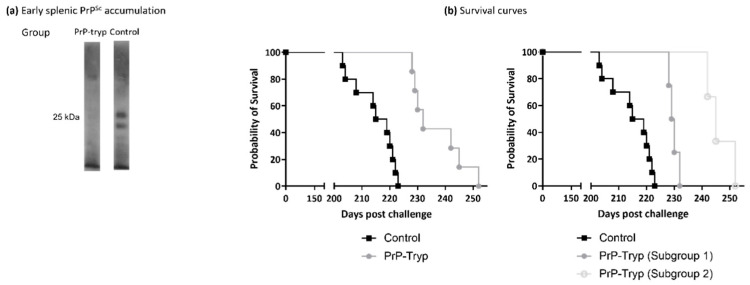
Immunization provides partial protection in the murine animal model. (**a**) Representative Western blotting of splenic homogenates enriched in PrP^Sc^ from PrP-tryp immunized and control mice, using 6H4. PrP-tryp immunized mice accumulate less splenic PrP^Sc^ at the early stages of disease (a total of four PrP-tryp immunized and four control immunized mice were sacrificed at early timepoints). (**b**) Survival curves indicate that PrP-tryp-immunized (N = 7) mice survive significantly longer than control (Ν = 11) mice (left panel, Log-rank (Mantel-Cox) test, *p* < 0.0001). Immunization seems to result in different levels of protection, as two distinct subgroups with different survival curves (Log-rank (Mantel-Cox) test, *p* = 0.0177) can be identified (right panel).

Spleens from mice sacrificed at the early timepoint were homogenized and enriched in PrP^Sc^. Splenic PrP^Sc^ content was estimated by Western blotting with SAL1 [31], a polyclonal anti-PrP serum (Figure 4a). At this early timepoint, mice immunized with the engineered trypanosomes had accumulated significantly less splenic PrP^Sc^, compared to control mice.

The remaining mice were kept under constant observation and euthanized when they reached the terminal stage, characterized by a significant weight loss accompanied by marked dyskinesia and/or paralysis. Accumulation of PrP^Sc^ in the brain was confirmed by Western blotting in brain homogenates, using monoclonal antibody 6H4, and no differences were detected (Supplementary Figure S1). The survival curves (Figure 4b, left panel), on the other hand, were significantly different (Log-rank (Mantel-Cox) test, *p* < 0.0001), with PrP-tryp-immunized mice surviving longer than control mice (236.9 ± 3.56 vs. 214.9 ± 2.37 days; two-tailed *t* test, *p* = 0.0004).

Interestingly, the PrP-tryp immunized mice could be further distributed into two subgroups: one with a shorter survival interval (229 ± 1.70), and a second with a longer one (246.3 ± 5.13). Difference in the mean survival intervals of the two subgroups was found to be statistically significant (two-tailed t test, *p* = 0.0016), as were the survival curves (Log-rank (Mantel-Cox) test, *p* = 0.0177). Although the antibody titer for each immune sera was not formally calculated, the three mice that survived longest also displayed the highest reactivity in flow cytometry experiments.

## 3. Discussion

Passive immunization against prion diseases has led to very encouraging results in different studies. However, such approaches are characterized by some inherent weaknesses that significantly reduce their potential for wider use. Passive immunization is transient and short-lived, thus requiring repeated administration of the protective antibodies for it to be maintained [32]. To efficiently treat prion diseases in a murine model, repeated antibody administrations (twice weekly for more than 350 days and once weekly afterwards) were required [12]. Moreover, for the antibody treatment to be efficient, it is of paramount importance to start within a rather short window of time, following exposure to the pathogen. That is not always feasible, especially if the subject is repeatedly exposed to the pathogen in the field, as may be the case for zoonotic prion diseases.

On the other hand, active immunization approaches, with the exception of mucosal immunization, were not equally successful. Generation of antibodies against the prion protein is unimpeded in PrP KO animals [27,33,34], but tolerance effects severely hamper active immunization in PrP-expressing animals [26,27]. PrP is almost ubiquitously expressed, including the thymus [35], and the low immune response against the prion protein when wild type animals are immunized could be associated with the deletion of precursors of Th and B cells that express T or B cell receptors that recognize prion protein epitopes. This, in turn, leads to a reduction of the repertoire of B cell precursors with PrP affinity, hampering development of a robust response upon immunization with the prion protein [26]. These tolerance effects against the prion protein in animals expressing the protein have been overcome, through the use of adjuvants, such as Freund’s adjuvant [16,17,18,19,36,37,38,39,40,41,42], alum [13,14,15], CPG oligonucleotide alone [20,26,43] or in combination with FA [44], cholera toxin [45], AdjuVac [23], Montanide IMS 1313 [46], Emulsigen-D [47,48] and/or the use of specific homologous or heterologous PrP epitopes [36,47,48], PrP fragments [20,26,38,41,44,46], or PrP monomeric [16,19,40] or multimeric [17,18,39,49,50,51] forms with the aim to present to the host immune system domains of the protein that are not exposed on membrane-bound PrP. To circumvent tolerance effects, without the use of adjuvants, we explored the use of trypanosomes, engineered to express a PrP peptide on their VSGs.

We chose to introduce the HFGNDWEDRYYRENMY peptide, corresponding to murine prion 139–152, flanked by three glycine residues on each side, which allow an added level of structural flexibility and facilitate expression of the peptide without severely compromising folding of the VSG. The prion peptide corresponds to a peptide located in the central part of the protein, which is believed to be important for conversion of PrP^C^ to PrP^Sc^ [52] and entails the murinized epitope of the anti-prion monoclonal antibodies 6H4 (D**W**EDRYYRE) [53] and SAF61 [54,55]. Proof of principle for the anti-prion efficiency of the 6H4 antibody was provided in a murine model of prion diseases, wherein transgenic expression of the antibody’s μ chain fully protected PrP^+/-^ mice upon challenge [56]. In a different set of experiments, SAF61 blocked conversion of the prion protein in in vitro disease models [54,55].

To generate the engineered trypanosomes, trypanosomes were transfected with several different plasmids, encoding for either VSG 117 or VSG2, tagged with the HA- or FLAG-tag, and the prion peptide at different locations within the glycoprotein. Following selection of the stably transfected clones, trypanosomes were checked for surface expression of the prion peptide by flow cytometry. Efficient expression was achieved only in one of the combinations tested, wherein the tag and the peptide were introduced at the most distant locations in VSG2. This low rate could be attributed to the rigorous quality control that VSGs undergo, resulting in proteasomal digestion of improperly folded VSGs, or alternatively, to VSG conformations that masked the prion peptides.

The prion peptide-expressing trypanosomes were formalin-fixed and used to immunize wild-type BALB/c mice. Sera after the second boost were able to recognize recombinant prion protein, as well as PrP^C^ and PrP^Sc^ from brain homogenates of terminally ill mice in Western blots. Moreover, immune sera recognized native PrP on the surface of PrP-overexpressing DT-40 cells, which is a strong indicator of therapeutic potential against TSEs [39], prompting us to perform a pilot bioassay. This robust immune response was achieved using a very basic immunization protocol, consisting of three i.p. administrations without the addition of adjuvants. The use of adjuvants has been associated with adverse reactions, and questions have been raised regarding their use in animals and humans [57,58]. Thus, trypanosomes could emerge as immunization platforms that eliminate the need for adjuvant use due to their high immunogenicity. The well-documented high immunogenicity of the Trypanosomes’ VSG coat is not only associated with the repetitive structure of its core units, but it has also been postulated that the VSG protein may lead to non-specific B cell activation [59]. In addition to the high immunogenicity, immunization with trypanosomes is very versatile, as diverse antigens can be incorporated in the VSG and the preparation of the antigens is extremely easy, fast and scalable, as it entails growing and fixing the antigen presenting trypanosomes, without the need for time-consuming purification steps.

Immunized mice were challenged i.p. with a mouse-adapted scrapie strain. Following peripheral challenge, PrP^Sc^ is amplified in the spleen and, as a result, it accumulates even at early timepoints. However, early splenic PrP^Sc^ accumulations were substantially reduced in mice immunized with the prion peptide-expressing trypanosomes, indicating a protective effect. The protective effect was further confirmed in the survival prolongation: mice immunized with the engineered trypanosomes survived significantly longer (~22 days) than control mice.

The prolongation of survival appears to be associated with the immunization efficiency. Mice immunized with the engineered trypanosomes could be further allocated into two subgroups, based on the survival prolongation. The longer-surviving mice also had the highest reacting sera against PrP-expressing DT-40 cells in flow cytometry. This finding indicates that optimization of the immunization protocol, for example via additional administrations of trypanosomes or through a mix of different prion peptide-expressing trypanosomes could enhance the protection provided. However, it must be noted that partial protection is a recurrent finding in active immunization protocols against prion diseases: with the exception of mucosal immunization protocols where most probably the antibodies generated prevent uptake of the pathogen by the host, full protection has not yet been achieved, although an immune response has been elicited [10]. Since efficient interactions between the antibodies and the prion protein can be achieved [39,54,55], and at least in some cases passive immunization approaches can provide almost full protection [12], maybe different aspects of the protocols, such as the antibody titers and the kinetics of antibody production, need to be addressed before full protection is achieved via active immunization. Our work hypothesis, corroborated by the finding that anti-PrP antibodies triggered by PrP-tryp immunization can bind to PrP on the cell surface of DT40 cells, is that the antibodies generated can bind to cellular PrP^C^ either in the periphery or the neurons and impede, at least in part, the interaction between host PrP^C^ and PrP^Sc^ introduced through the inoculum. Anti-PrP antibodies cannot completely block the interaction between PrP^C^ and PrP^Sc^, thus even in immunized mice the conversion of PrP^C^ to PrP^Sc^ takes place, albeit at a lower rate. Moreover, the rate of production of the anti-PrP antibodies in the PrP-tryp immunized animals slowly deteriorates, as the antigen is processed and the newly generated—through the conversion of PrP^C^ to PrP^Sc^—PrP^Sc^ antigens cannot trigger memory cells to initiate a robust secondary response. Eventually, PrP^Sc^ molecules reach a threshold whereby they outnumber the anti-PrP antibodies even in PrP-tryp immunized mice and the PrP^C^ to PrP^Sc^ conversion rate is elevated, reaching normal levels. The effect is that through active immunization, only a delay in disease progression can be achieved. Collectively, both the number of the antibodies elicited, as well as the specificity for given epitopes and their half-life may play a role, explaining why some of the immunized animals are less well protected compared to others and, so far, complete protection against prion diseases has not been achieved with active immunization protocols.

In order to secure full protection through active immunization, we need to maintain a constant concentration of antibodies with the required specificity for PrP for longer time frames. That could be achieved either through repeated boosts or via the identification of antigens that would lead to the production of memory cells with a specificity for antigens exposed by PrP^Sc^. Moreover, better understanding of the conversion mechanism and the critical domains involved, such as the recently identified R208-H140 hydrogen bond [60], would enable improved targeting of the antibodies.

Our findings indicate that trypanosomes could successfully function as a flexible platform for eliciting potent humoral immune responses without the use of adjuvants, even in cases where the antigen is not highly immunogenic, or tolerance effects impede activation of the immune system. This could contribute towards the development of efficient and safe immunoprophylactic approaches in prion and other diseases.

## 4. Materials and Methods

### 4.1. Generation of Expression Plasmids

Regarding the backbone for the generation of the expression plasmids, pUB39 plasmids already encoding for VSG 117 (MITat 1.4) tagged with HA at different positions or with VSG2 (MITat 1.2 or VSG 221) tagged with FLAG at different sites were used, as previously described [30]. Briefly, DNA fragments were generated using dedicated primers in a two-step assembly PCR approach. These DNA fragments encoded for the GGGHFGNDWEDRYYRENMYGGG prion peptide and were inserted in 4 VSG 117 and 11 VSG2 sites, while retaining expression of the HA or FLAG tag correspondingly. To improve fidelity, all PCR reactions were performed with hot start KOD polymerase (Takara Bio, Shiga, Japan). The DNA fragments produced were first digested with BamHI and HindIII (New England Biolabs, Ipswich, MA, USA), and then ligated to pUB39 plasmids (a generous gift from Dr. G. Cross, Rockefeller University). DH5a cells (Sigma Aldrich, St. Louis, MO, USA) were transformed with the plasmids and, following confirmation of the inserted sequence by Sanger sequencing, the plasmids were further amplified and purified (Qiagen, Germantown, MD, USA). Before transfection, plasmids were linearized by NotI digestion (New England Biolabs, Ipswich, MA, USA). A total of 34 plasmids were prepared.

### 4.2. Trypanosoma Brucei Growth and Transfection

The bloodstream-form trypanosomes derived from the Lister 427, expressing VSG427-2, were used throughout the experiments and grown in HMI-9 medium (produced in house) at 37 °C, 5% CO_2_. Trypanosomes were transfected with the Not I linearized plasmids using the Amaxa Nucleofector (Lonza, Visp, Switzerland) system. Briefly, 10^6^ trypanosomes were transfected with 10 μg of the plasmid, using setting x-001, then diluted 10×, and transferred into 24-well plates. Twenty-four hours later, the selection medium (Phleomycin, Invivogen, San Diego, CA, USA) was added, and cell survival was assessed by optical microscopy following Trypan blue (Sigma Aldrich, St. Louis, MO, USA) staining.

### 4.3. Flow Cytometry

Surface expression of the FLAG, HA and PrP on the transfected trypanosomes was assessed by flow cytometry, using untransfected trypanosomes as negative controls. For FLAG and HA expression, approximately 10^6^ cells were pelleted and incubated at 4 °C for 15 min with serum implemented with anti-FLAG M2 (FITC-conjugated, Sigma Aldrich, St. Louis, MO, USA), or with anti-HA (PE-conjugated, Miltenyi Biotec, Bergisch Gladbach, Germany), at a final concentration of 1 μg/mL. Cells were then rinsed, resuspended in medium and the fluorescence was estimated with a BD Biosciences FACSCalibur.

To assess surface expression of the PrP peptide on the transfected trypanosomes, 10^6^ cells were pelleted and resuspended in medium containing the monoclonal anti-PrP antibody 6H4 (ThermoFisher Scientific, Waltham, MA, USA) at a final concentration of 1 μg/mL at 4 °C for 15 min. Cells were then rinsed, pelleted and resuspended in medium containing FITC-labeled goat anti-mouse IgG (Miltenyi Biotec, Bergisch Gladbach, Germany) at a final concentration of 1 μg/mL at 4 °C for an additional 15 min. Cells were then rinsed, pelleted, resuspended in medium and their fluorescence was estimated by flow cytometry.

The ability of the generated immune sera to recognize PrP expressed on the cell surface was also assessed by flow cytometry. In this case, DT-40 cells stably transfected to express the full length murine PrP on the cell surface were used. Briefly, 10^6^ DT-40 cells were pelleted and incubated with sera from the immunized mice (diluted 1:200 *v*/*v* in medium), with the monoclonal anti-PrP antibody 6H4 (positive control, 1 μg/mL final concentration in medium), or with the medium alone (negative control) at 4 °C for 15 min. Cells were then rinsed and pelleted before being resuspended in medium containing FITC-labeled goat anti-mouse IgG (Miltenyi Biotec, 1 μg/mL in medium) at 4 °C for 15 min. Following rinse and resuspension in medium, the fluorescence was estimated by flow cytometry. Flow cytometry data were analyzed with FlowJo software (BD Biosciences, Franklin Lakes, NJ, USA).

### 4.4. Immunization and Bioassay

All animal studies were performed in accordance with institutional and national regulations. The protocol was approved by the local veterinary services (protocol numbers: 320190/2813 and 386435/3393).

In total, 26 female BALB/c mice, acquired from Charles Rivers laboratories, were used for the studies. Mice were housed in a biosafety level 3 facility with ad libitum access to food and water. Temperature was set at 21 °C, relative humidity at 30% and the day cycle at 12 h.

Following a week of acclimatization at the facility, the mice were immunized with the inactivated trypanosomes. To inactivate trypanosomes, cells were treated with 3% *v*/*v* formaldehyde in phosphate buffered saline (PBS, ThermoFisher Scientific) for 1 h at room temperature. The immunization scheme consisted of pthree i.. administrations of inactivated trypanosomes (10^7^ cells each in 100 μL PBS) with 45-day intervals. Sera were collected from all the mice the day before immunization started (pre-immune sera), and then 7 days after the second and the third trypanosome administration, via submandibular bleeding [61]. Experimental groups received genetically engineered trypanosomes expressing the prion protein peptide (PrP-tryp immunized group), while the control group received wild type trypanosomes (control group).

Four weeks after the immunization protocol was completed, mice were challenged i.p. with a mouse-adapted scrapie strain (RML [62], 10^3.5^ × LD_50_). Mice were monitored daily to assess disease progression. Early symptoms included abnormal gait and minor ataxia, followed by loss of motor coordination, dyskinesia, kyphosis and eventually weight loss and paralysis. Mice were euthanized by cervical dislocation under isoflurane anesthesia either at an early timepoint, before the appearance of any disease symptoms (day 50 post challenge) or at the terminal stage. The terminal stage was defined by severe weight loss with dyskinesia or paralysis. The brain and spleen were collected, snap frozen and stored at −80 °C until further analysis.

### 4.5. Western Blotting

Recombinant murine PrP or tissue (brain or spleen) homogenates were prepared and resolved by SDS-PAGE, as previously described. Briefly, 1 μg of protein or 2.5 mg tissue equivalent with or without PrP^Sc^ enrichment [63] were resolved on 12% polyacrylamide gels and electro-transferred on PVDF membranes (Millipore, Burlington, MA, USA). The membranes were blocked with blocking buffer (5% non-fat dry milk in PBS, supplemented with Tween20 (Sigma, 0,1% *w*/*v*, PBST) at room temperature for 1 h. Sera from the immunized mice (1:200 *v*/*v* in blocking buffer), a polyclonal anti-PrP serum (SAL1 [31], diluted 1:5000 *v*/*v* in blocking buffer) or 6H4 (0.2 μg/mL in blocking buffer) were then added at 4 °C overnight. Following washes with PBST, HRP-conjugated rabbit anti-mouse IgG was added (ThermoFisher Scientific, 0.1 μg/mL in blocking buffer) at room temperature for 1 h. Following washes with PBST, proteins were visualized with the ECL reagent (ThermoFisher Scientific).

## Data Availability

Not applicable.

## Supplementary Materials

The following supporting information can be downloaded at: https://www.mdpi.com/article/10.3390/ijms231810629/s1.
