# Supplementary material for: Immunization with Genetically Modified Trypanosomes Provides Protection against Transmissible Spongiform Encephalopathies
Source: Int J Mol Sci. 2022 Sep 13;23(18):10629. doi: 10.3390/ijms231810629 (PMC9503410; doi:10.3390/ijms231810629)
Supplement: Supplementary file 1 [file ijms-23-10629-s001.zip › ijms-1893665-supplementary.pdf]

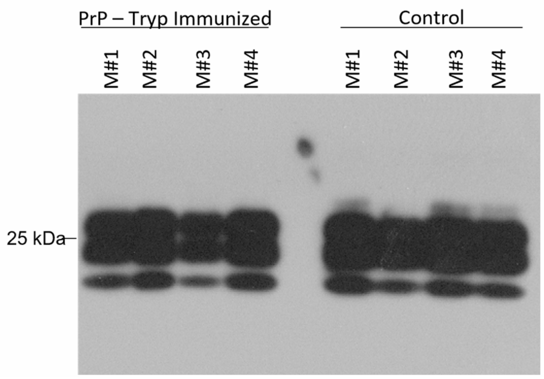

Figure S1. PrP^{Sc} in brain homogenates from terminally ill mice. A representative western blot of brain homogenates (2.5mg brain equivalent) enriched in PrP^{Sc} from PrP-tryp immunized and Control mice, using monoclonal antibody 6H4. The band intensities were estimated for all brain homogenates (7 from PrP-Tryp immunized and 10 from Control immunized animals) using ImagJ software and no statistically significant difference was detected between the two groups (Unpaired T test, P= 0.6611).
